# The Likelihood of Experiencing Relative Poverty over the Life Course

**DOI:** 10.1371/journal.pone.0133513

**Published:** 2015-07-22

**Authors:** Mark R. Rank, Thomas A. Hirschl

**Affiliations:** 1 George Warren Brown School of Social Work, Washington University, St. Louis, Missouri, United States of America; 2 Department of Development Sociology, Cornell University, Ithaca, New York, United States of America; IFIMAR, UNMdP-CONICET, ARGENTINA

## Abstract

Research on poverty in the United States has largely consisted of examining cross-sectional levels of absolute poverty. In this analysis, we focus on understanding relative poverty within a life course context. Specifically, we analyze the likelihood of individuals falling below the 20th percentile and the 10^th^ percentile of the income distribution between the ages of 25 and 60. A series of life tables are constructed using the nationally representative Panel Study of Income Dynamics data set. This includes panel data from 1968 through 2011. Results indicate that the prevalence of relative poverty is quite high. Consequently, between the ages of 25 to 60, 61.8 percent of the population will experience a year below the 20^th^ percentile, and 42.1 percent will experience a year below the 10^th^ percentile. Characteristics associated with experiencing these levels of poverty include those who are younger, nonwhite, female, not married, with 12 years or less of education, or who have a work disability.

## Introduction

In recent years there has been an increasing amount of attention focused on the issue of income inequality in the United States [1]. Since the mid 1970's, research has indicated that income inequality has gotten wider. For example, Saez finds that in 1975 the top 10 percent of the population earned approximately 33 percent of the overall income, whereas by 2012 the figure was slightly over 50 percent [2]. Likewise, the Gini coefficient of overall inequality has been rising in the United States. In 1975 it was .397. By 2013 it had risen to .476 [3].

While highly informative, much of this research has been limited by cross-sectional data and methods. As a result, we know relatively little about the dynamics of income inequality. In other words, to what extent is there mobility in and out of the top and bottom of the income distribution? Given widening levels of inequality, mobility dynamics are of increasing material significance.

In addition, much of the focus on income inequality has been directed to the top of the income distribution [4, 5]. There has been a lack of attention to the bottom part of the income distribution to determine what the life course dynamics of relative poverty are.

In this paper we focus on the likelihood of individuals falling below the 20^th^ percentile and 10^th^ percentile of the income distribution between the ages of 25 and 60 as life course indicators of experiencing poverty and extreme poverty. Our focus is upon the extent that individuals experience relative poverty over time, the life course timing of poverty, whether this experience is long-term or acute, and the characteristics of those who are more likely to experience relative poverty.

### Background

There are a number of ways in which to measure poverty [6]. However, a key distinction is between an absolute versus a relative approach [7]. Absolute measures of poverty basically define a minimum threshold for living conditions, and individuals who fall below that threshold are considered poor. Consequently, there is a line that is drawn in income, and those falling below that income line are counted as impoverished. The official U.S. federal poverty line is one example of an absolute approach to measuring poverty.

On the other hand, poverty can be measured in a relative sense. For example, individuals who fall into the bottom 20 percent of the income distribution might be considered poor. Alternatively, the poor could be defined as those whose incomes fall below 50 percent of the population’s median income.

The majority of longitudinal studies of poverty have used absolute metrics, and in particular the federal poverty line [8, 9]. Although this research has revealed a number of important insights, it has also been hampered by the use of an absolute approach, and specifically, the use of the federal poverty line.

Drawbacks to the official poverty line include the fact that it is based on the food needs of families, and has therefore not been affected by the rising costs of other essential goods and services (e.g. housing, child care, transportation, etc.). The multiplier of three times the economy food plan no longer represents the fundamental needs of families.

Second, when the poverty line was introduced in 1964, it represented approximately one half of median income. By 2013, it constituted approximately 30 percent of median income. Consequently, the meaning of poverty has shifted in a relative sense, but the poverty line itself has not kept up.

Third, there is considerable debate over where the poverty line should be drawn. Some argue it is set too low, while others argue that it is set too high. Ultimately, where the poverty line is actually drawn has an arbitrary element to it.

Finally, the absolute approach to measuring poverty tells us little about the extent of inequality in the society. A longitudinal relative approach, on the other hand, gives us a sense of the extent of income inequality at the bottom end of the income distribution.

The present study of relative poverty therefore addresses a gap within the research literature, a gap that has arguably become more important to investigate given the emphasis upon income inequality. Whereas the logic of absolute poverty is derived from a needs standard, relative poverty measures relative depravation, a concept that has greater salience in the context of rising inequality [10].

In addition, a primary focus of research on income mobility and poverty has been intergenerational mobility, and proceeds by measuring income over two periods of generally three to five years [11–14]. We are critical of this approach because it truncates the observation of income, obviating the fluid character of economic fortune across the American life course [4, 9]. Therefore we endeavor to organize this investigation of relative poverty dynamics in the United States using a life course approach.

The concept of the life course has had a long and distinguished history within the social sciences. It has proven to be an extremely helpful tool in thinking about the manner in which individual lives unfold. The term itself refers to “social processes extending over the individual lifespan or over significant portions of it, especially [with regard to] the family cycle, educational and training histories, and employment and occupational careers” [15].

The life course approach has been applied to understanding the extent of absolute poverty within the lifetimes of Americans. The work of Rank and Hirschl has demonstrated that the life course risk of absolute poverty and economic insecurity is quite high when compared to the cross-sectional risk. For example, between the ages of 25 and 60, 54 percent of the population will experience at least one year in poverty (150 percent below the federal poverty line). In addition, 79 percent of Americans will experience a year of economic insecurity, which includes either using a social welfare program, encountering poverty, or the head of household experiencing unemployment [9].

A gap in the life course research is that there has not been a study that has examined the likelihood of individuals experiencing various levels of relative poverty. In this article we focus on the chances that Americans will fall below the bottom 20^th^ percentile of the income distribution as well as the bottom 10^th^ percentile.

## Materials and Methods

### Data Set

In order to assess the life course dynamics of relative poverty over time, we utilize the Panel Study of Income Dynamics (PSID). The PSID began in 1968 as an annual panel survey (biennial after 1997) and is nationally representative of the nonimmigrant U.S. population. The longest running panel data set in the United States, the PSID gathers extensive information regarding household income, making it uniquely suited for this study. The PSID initially interviewed approximately 4,800 U.S. households in 1968, which included detailed information on roughly 18,000 individuals within those households. The PSID has since tracked these individuals, including children and adults who eventually broke off from their original households to form new households (e.g., children leaving home, separations, divorce). Thus, the PSID is designed so that in any given year the sample is representative of the entire nonimmigrant U.S. population (for detailed information regarding the PSID sample and its representativeness, see [16–19]).

Throughout the analysis we employ the individual sampling weights to ensure that the PSID sample accurately reflects the overall U.S. population. Specifically, we utilize the weights assigned to individuals for each given wave to take advantage of the PSID practice of periodically adjusting the weights to account for nonresponse bias [20].

We utilize both the household and individual levels of information from the initial wave of 1968 through 2011. Consequently, we draw upon 44 years of longitudinal information, which translates into several hundred thousand person-years of information embedded in the analysis. As noted earlier, after the 1997 wave, the PSID began interviewing households every other year. A second change occurring in 1997 was that the PSID sample size was reduced for cost management reasons. The original core sample was reduced from approximately 8,500 in 1996 to 6,168 in 1997 [16]. As noted above, the sample weights are used throughout to ensure that the sample continues to represent the overall population and that the reduction in sample size does not bias our estimates [21].

Overall family income is the measuring stick used to determine levels of poverty experienced. This is defined as taxable income of head of household and spouse, taxable income of other family members of the household, and transfer income of head, spouse, and others. The PSID questionnaire includes a lengthy set of income questions designed to recover multiple forms of taxable income sources.

### Life Table Technique

In detailing the life course patterns of poverty dynamics over time, we rely upon the life table as our major analytical technique. Life tables are a concise method for describing how the odds of experiencing a specific event change as individuals age over time. The life table is most closely associated with biological and demographic studies of mortality, but can be easily applied to estimate the occurrence of other events as well [22, 23].

In this analysis, our time intervals comprise each year (or two) that an individual ages. During that year, one can calculate the probability of an event occurring (in this case, poverty) for those who have yet to experience the event. Once the event has occurred, the individual is no longer at risk and therefore exits the life table. Based upon these age specific probabilities, the cumulative probabilities of an event occurring across the life course can be calculated. These cumulative probabilities form the core of our analysis.

One of the consequences (and potential advantages) of this approach is that period effects are smoothed out within and across age intervals. For example, some of the approximately 18,200 individuals in the 25 year old group experienced their 25^th^ year in 1968, some in 1975, some in 1990, and so on. The advantage of this approach is that historical effects such as recessions do not unduly affect any particular age group or the hypothetical cohort as a whole (which can happen if one uses only one point in time to construct a life table).

Individuals may contribute anywhere from 1 to 36 person-years within the life table. For example, a woman within the PSID study who turned 25 in 1975 and then in 1979 experienced a year of poverty would have contributed five person years within our analysis. In this case, she would be included in the estimates for ages 25, 26, 27, 28, and 29.

Life tables are calculated for those falling below the bottom 20^th^ percentile in terms of household income, as well as below the bottom 10^th^ percentile. These levels have served to represent poverty and extreme poverty in prior research [3], and we refer to them as such in our results section. The percentiles were calculated for each year of the PSID for individuals aged 25 to 60. For 2010 (reported year of income by the 2011 data wave), the cutoff point for the 20^th^ percentile was $25,368, and for the 10^th^ percentile it was $14,447. Over the study period 1968 to 2011, the 20th percentile averaged 49 percent of median household income, and the 10th percentile averaged 30 percent of median household income (data and calculations available from the authors upon request).

To measure chronicity for each of these levels, life tables are calculated for individuals that experience one or more years, two or more years, three or more years, four or more years, five or more years, and ten or more years. Finally, calculations are made for consecutive versus total years to identify spell length within each of these levels.

## Results

**Fig 1:**
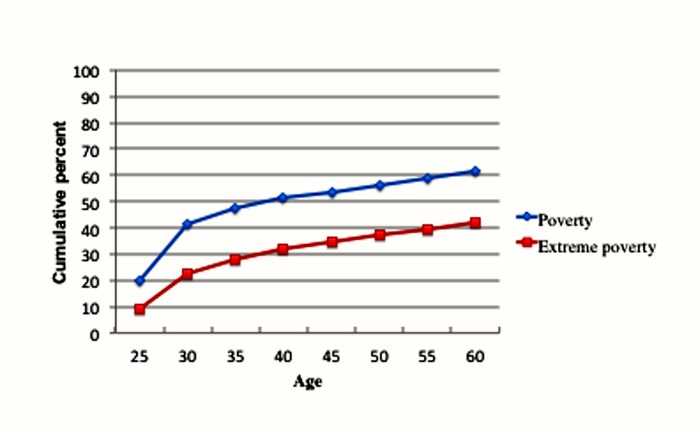
Cumulative percentage of American adults experiencing poverty and extreme poverty by age.

Table 1 contains the cumulative percentage of adults experiencing poverty (defined as falling below the bottom 20^th^ percentile of the income distribution) between the ages of 25 and 60 (also shown in Fig 1). We can see that by age 30, 41.6 percent of the population has experienced a year of poverty, by age 40 the percentage is 51.2, by age 50 it is 56.5 percent, and by age 60, 61.8 percent of the American population will have experienced a year of poverty. Consequently, these results indicate that six out of ten Americans will encounter a year in poverty as defined by occupying the bottom 20^th^ percentile of the income distribution.

**Table 1:** Cumulative Percentage of American Adults Experiencing Poverty by Age. (Standard Errors in Parentheses)

	Bottom of Income Distribution	
Age	20 percent	10 percent
25	19.9 (2.7)	9.2 (1.9)
30	41.6 (3.4)	22.7 (2.9)
35	47.7 (3.6)	27.7 (3.3)
40	51.2 (3.8)	31.8 (3.6)
45	53.9 (3.9)	34.6 (3.9)
50	56.5 (4.2)	37.3 (4.3)
55	59.1 (4.6)	39.5 (47)
60	61.8 (5.1)	42.1 (5.4)

We can also see the cumulative percentages of the population that will experience a year of extreme poverty (define as falling below the bottom 10^th^ percentile of the income distribution). By age 30, 22.7 percent of Americans have experienced at least one year of extreme poverty, by age 40 the percentage is 31.8, by age 50, 37.3, and by age 60, 42.1 percent of the population will have encountered a year in which their household income fell into extreme poverty. These results indicate that there is a substantial amount of downward income mobility at these percentile levels.

Fig 1 displays the graphical plotting of these percentages. We can see that there is a rapid increase in the cumulative percentage of the population experiencing poverty and extreme poverty between ages 25 and 35. After age 35, the poverty and extreme poverty lines begin to flatten out.

Table 2 displays the overall number of years of poverty and extreme poverty experienced between the ages of 25 to 60. The top panel contains the total number of years, whereas the bottom panel shows the consecutive number of years. While 61.8 percent of the population will experience at least 1 year of poverty, 45.0 percent will experience at least 2 years, 34.8 percent will experience 3 or more years, 29.4 percent will encounter 4 or more years, 24.9 percent will experience 5 or more years, and 11.9 percent will encounter 10 or more years. With respect to experiencing extreme poverty, the percentages are 42.1, 25.8, 19.0, 14.6, 11.4 and 4.1.

**Table 2:** Years of Poverty Experienced Between the Ages of 25 to 60. (Standard Errors in Parentheses)

	Bottom of Income Distribution	
Years of Poverty	20 percent	10 percent
**Total Years**		
1 or more	61.8 (5.1)	42.1 (5.4)
2 or more	45 (5.4)	25.8 (5.0)
3 or more	34.8 (5.4)	19.0 (4.8)
4 or more	29.4 (5.4)	14.6 (4.5)
5 or more	24.9 (5.4)	11.4 (4.0)
10 or more	11.9 (4.2)	4.1 (2.7)
**Consecutive Years**		
1 or more	61.8 (5.1)	42.1 (5.4)
2 or more	38.2 (5.1)	20.5 (4.6)
3 or more	27.4 (5.0)	13.2 (4.1)
4 or more	21.1 (4.7)	9.1 (3.6)
5 or more	15.3 (4.1)	5.9 (2.7)
10 or more	5.6 (3.0)	1.5 (1.5)

The bottom panel of Table 2 displays the percentage of the population experiencing various consecutive years at particular income levels. These percentages are lower than the total number of years. Thus, 5.6 percent of the population will experience 10 consecutive years in poverty, while 1.5 percent will do so in extreme poverty. Overall, the results in Table 2 confirm the proposition that experiencing relative poverty is prevalent in the United States, and that it often occurs over relatively short periods of time.

**Fig 2:**
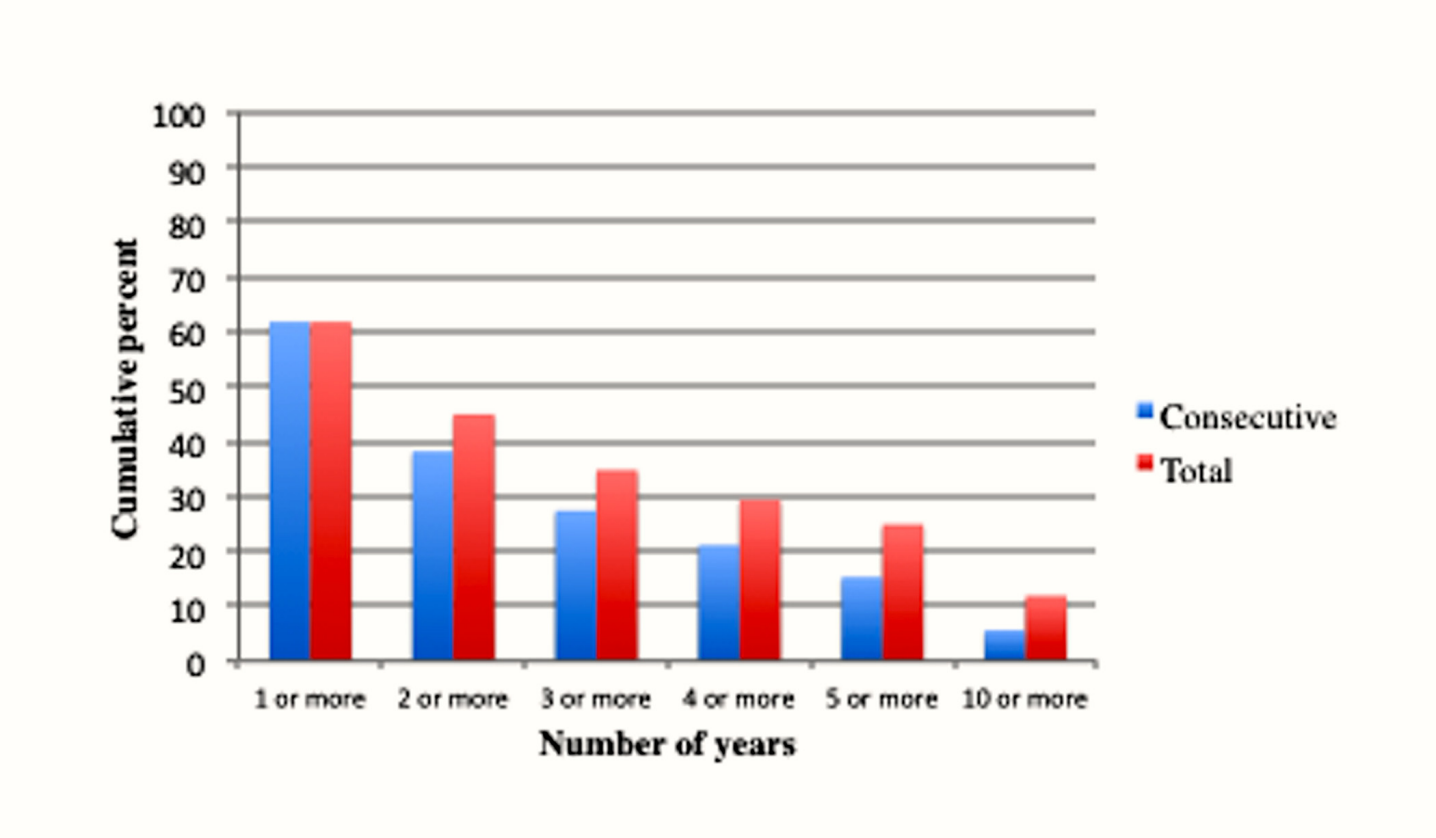
Years of poverty experienced between ages 25 to 60.

**Fig 3:**
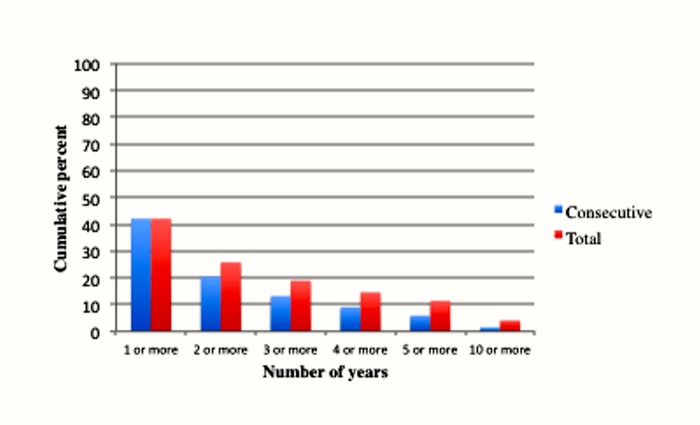
Years of extreme poverty experienced between ages 25 to 60.

In Figs 2 and 3 we present these percentages as bar graphs. Fig 2 compares the total number of years and consecutive number of years for poverty. Fig 3 shows the same percentages for extreme poverty. We can see that a greater percentage of the population experience total number of years than consecutive number of years at each length of time (the rates are obviously identical for 1 or more years). In addition, we can compare the difference between poverty and extreme poverty in terms of their corresponding lengths of time. Clearly individuals are more likely to encounter poverty than they are extreme poverty at each of the specific time lengths.

In Table 3 we look at the occurrence of poverty over separate 10 year age intervals across the life course. We therefore start the life table analyses at the beginning of each 10 year age interval. In general, research has shown that poverty is more likely to occur during the earlier and later periods of the prime working years [3]. We can see that between the ages of 25 and 34, 46.9 percent of the population will experience at least one year of poverty. Between the ages of 35 and 44, the percentage is 31.1, 28.8 percent between 45 and 54, and 38.9 percent between 55 and 64. Thus, there is a U shaped pattern to the risk of experiencing poverty by age categories.

**Table 3:** Cumulative Percentage of American Adults Experiencing Poverty Across Age Categories. (Standard Errors in Parentheses)

	Bottom of Income Distribution	
Age Category	20 percent	10 percent
25–34	46.9 (3.6)	26.9 (3.2)
35–44	31.1 (3.5)	18.8 (3.0)
45–54	28.8 (3.6)	18.0 (3.1)
55–64	38.9 (4.5)	22.9 (3.8)

We find the same pattern with respect to extreme poverty. Between the ages of 25 and 34, 26.9 percent of Americans will encounter a year of extreme poverty, 18.8 percent between 35 and 44; 18.0 percent between 45 and 54; and 22.9 percent between 55 and 64.

In Table 4 we present a multivariate analysis predicting the occurrence of the two levels of poverty. The logistic regression odds ratios for each independent variable are found in the table. Age is entered as a continuous variable, whereas the other variables are dichotomous. They include race (nonwhite/white), gender (female/male), marital status (not married/married), education (12 years or less/greater than 12 years) and work disability status (work disability/no work disability). The unit of analysis for the model is person-years.

**Table 4:** Logistic Regression Model Odds Ratios Predicting the Occurrence of Poverty Between the Ages of 25 and 60.

Variables	10 percent	20 percent
Age	.97 ^b^	.98 ^b^
Nonwhite	2.70 ^b^	2.93 ^b^
Female	1.20 ^b^	1.24 ^b^
Not Married	6.99 ^b^	8.03 ^b^
LE 12	2.35 ^b^	2.43 ^b^
Disability	2.83 ^b^	2.62 ^b^
N ^a^	174,927	174,927

^a^ "N" refers to the sample size.

^b^ Statistically significant at the .001 level

To assess the extent that the independent variables are collinear, two diagnostics were used. First, a linear model was estimated along with variance inflation factors (VIF) for each of the independent variables. All VIFs were found to be less then 2.0, hence well below the value 10.0 where multicollinearity is indicated [24]. Second, principal component factors were extracted from the independent variable matrix, and the condition indexes (CI) were inspected. The largest CI was found to be 12.3, again below the value 30 indicating multicollinearity [24]. On the basis of these two diagnostic tests, available from the authors upon request, we would argue that the estimation results presented in Table 4 are unlikely to be confounded by multicollinearity.

We can see that all independent variables are significantly associated with experiencing poverty and extreme poverty. Those who are younger, nonwhite, female, not married, with 12 years or less of education, and who have a work disability, are significantly more likely to encounter a year of poverty or extreme poverty.

## Discussion

In this article, we examine the life course odds of experiencing relative poverty between the ages of 25 and 60. Our measure of relative poverty is whether individuals reside in households in which their annual income falls below the 20^th^ income percentile. We also include a measure of extreme poverty where individuals fall below the 10^th^ income percentile.

Our results indicate that the occurrence of relative poverty is fairly widespread. Between the ages of 25 and 60, 61.8 percent of the population will experience at least one year of poverty, whereas 42.1 percent will experience extreme poverty. Furthermore, 24.9 percent of the population will encounter five or more years of poverty, and 11.4 percent will experience five or more years of extreme poverty.

As is the case with research on absolute poverty dynamics, a predominate pattern is that individuals are often likely to experience one or two years of poverty, and then rise out of poverty, with perhaps an additional spell down the road. Consequently, much of the poverty dynamics is characterized by a fluid movement into and out of poverty.

Poverty is also likely to occur across various 10 year age categories. Between the ages of 25 and 34, 46.9 percent of the population experienced poverty; between 35 and 44, the percentage is 31.1; 28.8 percent between 45 and 54; and 38.9 percent between 55 and 64.

The variables associated with a greater risk of poverty are those that have been found to be significant in prior research [25]. Consequently, those who are younger, with less education, having a work disability, being not married, nonwhite, and female are all characteristics that increase the odds of both poverty and extreme poverty.

The article has several strengths and weaknesses. First, the longitudinal study design avoids the limitation of truncated income observation, an important advantage to the extent that there is substantial annual variation in household income. In the present study, household income is observed for each year of the study, and the study findings reflect observed income over 36 years of the life course between ages 25 and 60. Second, life table techniques are used to compute lifetime income attainment, and these techniques yield robust life course estimates.

Weaknesses include a lack of sufficient sample size representing the U.S. immigrant population, and failure to describe and test a causal explanation related to the study objectives. Our aim, however, was not to identify causality related to bottom-level income, but rather to describe its pattern with respect to time.

In conclusion, our findings reinforce earlier work looking at the life course dynamics of absolute poverty [9, 26]. That research demonstrated that the life course odds of experiencing poverty were quite high. For example, between the ages of 25 and 60, 54 percent of the population will experience at least one year below 150 percent of the official U.S. poverty line [9]. Our findings in this article confirm the widespread prevalence of relative poverty within the population as well. Relative poverty is an economic condition that will strike the majority of Americans.

In addition, just as there is a great deal of fluidity at the top of the income distribution (70 percent of the American population will experience at least one year in the top 20^th^ percentile of the income distribution; [4]), so too is there substantial fluidity at the bottom of the income distribution. Taken together, these findings indicate that across the American life course there is a large amount of income volatility. Rather than a rigid class structure, the top and bottom ends of the income distribution are fairly porous. This finding provides an interesting and important caveat to the overall story of rising levels of income inequality across the past 40 years.
